# Enhancement of Bioactive Compounds and Antioxidant Properties in *Codonopsis pilosula* Through Extrusion Processing and Development of Codonopsis-Oat Powder

**DOI:** 10.3390/foods14203485

**Published:** 2025-10-13

**Authors:** Yuxuan Jia, Tie Jin

**Affiliations:** College of Agricultural, Yanbian University, Yanji 133002, China; jyx15145366099@163.com

**Keywords:** *Codonopsis pilosula*, twin-screw extrusion, physicochemical properties, antioxidant activity, product development

## Abstract

*Codonopsis pilosula*, a valuable traditional herb, is rich in bioactive compounds like polysaccharides and phenolics. However, conventional processing methods may limit its functional properties and application in modern food industries. Extrusion processing, as an efficient and versatile technology, offers a promising approach to enhancing the bioactivity and utilization of botanical materials. In this study, *Codonopsis pilosula* was enhanced through extrusion processing. The results demonstrated that extrusion under the optimal conditions (screw speed of 250 rpm, moisture content of 20%, and barrel temperature of 131 °C) significantly enhanced the properties of *Codonopsis pilosula*. Specifically, the polysaccharide content increased from 244.41 to 271.00 mg/g, and the water solubility index rose markedly from 12.99% to 40.79%. Concurrently, a significant improvement in antioxidant activity was observed, with the hydroxyl radical scavenging rate increasing from 52.89% to 69.27% and the DPPH radical scavenging rate from 60.43% to 67.35%. Based on the optimized extrusion conditions, a Codonopsis oat flour was developed. Through orthogonal experiments, the optimal formulation ratio was identified, resulting in a flour product with moderate color and viscosity, a distinctive aroma, and a maximum sensory score of 88.7. These results demonstrate that extrusion is a viable approach for enhancing the functional properties of *Codonopsis pilosula*, providing a theoretical basis for its application in food processing lines and the development of functional foods.

## 1. Introduction

*Codonopsis pilosula* (or Dangshen), the dried root of a species within the *Codonopsis genus*, has been traditionally used in Chinese medicine to alleviate symptoms such as poor appetite, fatigue, and asthma [1,2]. Its potential for integration into modern diets was underscored in 2020 when it was included in the official list of substances approved for both medicinal and food purposes by the National Health Commission and the State Administration for Market Regulation of China [3]. The health-promoting effects of *Codonopsis pilosula*, which include protecting myocardial cells [4], enhancing immunity [5,6], protecting gastrointestinal mucosa, exhibiting antitumor potential [7], anti-aging effects [8], anti-fatigue, and regulating blood glucose [9,10], are primarily attributed to its rich profile of bioactive compounds, such as polysaccharides, flavonoids, and phenolics [11]. However, the direct consumption of the raw root poses several challenges, including poor palatability, limited bioavailability of its active components, and low convenience, which hinder its broader application in food products. Furthermore, conventional processing methods are often time-consuming and may lead to the formation of undesirable compounds [12,13], further limiting their suitability as a food ingredient. Therefore, developing efficient and mild processing technologies is crucial to unlock the full potential of *Codonopsis pilosula* as a functional food, enhancing the accessibility of its beneficial compounds and improving its overall acceptability.

The saccharides of *Codonopsis pilosula* can be primarily categorized into monosaccharides, oligosaccharides and polysaccharides. Monosaccharides mainly include glucose, rhamnose, fructose and galactose, while oligosaccharides and polysaccharides are composed of monosaccharides and their derivatives, exhibiting complex structural compositions [14,15]. Wu et al. [16] extracted and isolated a neutral polysaccharide, COP-W1, from *Codonopsis pilosula*, which exhibited significant antioxidant activity, indicating its potential as a natural antioxidant. Mei et al. [17] significantly improved the polysaccharide extraction rate and better retained the antioxidant activity of Codonopsis polysaccharides using solid-state fungal fermentation technology. Yang et al. [18] identified the main monosaccharide components of *Codonopsis pilosula* polysaccharides using gas chromatography and demonstrated their significant inhibitory effects on α-amylase and α-glucosidase. Due to their antioxidant activity, ability to maintain intestinal microbiota balance, and immune-enhancing properties, *Codonopsis pilosula* polysaccharides are increasingly being applied in health foods.

Current research on the pharmacological effects of *Codonopsis pilosula* has mainly focused on its antioxidant activity, ability to enhance immunity, and potential to lower blood sugar levels. Codonopsis polysaccharides have been shown to reduce cancer cell survival rates [19], enhance antioxidant enzyme activity, activate cellular oxidative defense mechanisms [20], promote cell proliferation, alter phagocytic ability, enhance immune activity [21,22], and lower blood sugar levels [23].

However, the majority of this research has been conducted on extracts from the unprocessed root of *Codonopsis pilosula*. Studies investigating multi-stage variable-temperature extrusion processing of *Codonopsis pilosula* have not yet been reported. The twin-screw extruder functions as a continuous processing unit that integrates multiple unit operations, including transportation, mixing, heating, pressurization, shearing, cooking, and shaping [24]. The principle of twin-screw extrusion technology involves the direct conversion of raw materials into desired products utilizing high temperature, high pressure, and high shear force. As an emerging food processing technology, twin-screw extrusion provides notable advantages, including high energy efficiency, rapid processing capabilities, wide applicability, environmental sustainability, precise control over process parameters, and high productivity. This technology employs intense physical forces to induce a series of physical and chemical changes within the material inside the barrel, consequently altering the internal structure and chemical composition of products. While high temperature significantly impacts the extrusion process, the short residence time within the barrel substantially minimizes the degradation of heat-labile nutrients [25]. Simultaneously, the combined effect of high temperature and pressure effectively inactivates most putrefactive and pathogenic microorganisms, while instantaneous moisture vaporization contributes to achieving long-term preservation [26,27]. Following extrusion, the material subjected to high shear force experiences cell wall disruption, facilitating the release of nutrients and significantly enhancing the extraction yield of soluble compounds [28]. Ying et al. [29] demonstrated that extruded red ginseng exhibits superior water solubility compared to unprocessed samples. Extrusion processing significantly increased the acidic polysaccharide and total polysaccharide content of red ginseng. Furthermore, the DPPH radical scavenging activity and reducing power of the extruded red ginseng were markedly enhanced.

This study utilized *Codonopsis pilosula* powder as the raw material. Twin-screw extrusion processing was performed under varying conditions of screw speed (200, 250, 300 rpm), material moisture content (18%, 20%, 22%) and barrel temperature (115, 130, 145 °C). The primary objectives were to investigate the effects of extrusion operating parameters on the properties of *Codonopsis pilosula* and to establish a theoretical foundation for its future application in twin-screw extrusion technology. Furthermore, the extruded *Codonopsis pilosula* powder served as the base material for the development of a Codonopsis-oat powder product. This work provided direction for the multi-faceted utilization of extruded *Codonopsis pilosula* powder and offered valuable reference information for the deep processing of *Codonopsis pilosula*.

## 2. Materials and Methods

### 2.1. Materials

Codonopsis powder purchased from Yanji, Jilin Province, China. Anhydrous ethanol (Tianjin Kemiou Chemical Reagent Co, Ltd., Tianjin, China), sulfuric acid, sodium hydroxide, phenol, sodium nitrite, potassium persulfate, ferrous sulfate, hydrogen peroxide (Sinopharm Chemical Reagent Co, Ltd., Shanghai, China). Rutin (≥98%), lobetyolin (≥98%), 1,1-diphenyl-2-picryl-hydrazyl radical (DPPH) (≥98%), 2,2′-Azinobis-(3-ethylbenzthiazoline-6-sulphonate) (ABTS) (≥98%), gallic acid, Folin–Ciocalteu reagent (Shanghai Yuanye Bio-Technology Co, Ltd., Shanghai, China). methanol (HPLC grade), acetonitrile (HPLC grade), sodium carbonate (Shanghai Macklin Biochemical Technology Co, Ltd., Shanghai, China). All other chemicals were of analytical grade unless otherwise specified.

### 2.2. Preparation of Double-Screw Extrudate of Codonopsis pilosula

Dried *Codonopsis pilosula* roots were ground using a mill and sieved through an 80-mesh screen. The prepared powder was then processed using a DSE-30 model co-rotating twin-screw extruder. The extruder was operated under the following constant parameters: feed rate of 100 rpm, die nozzle diameter of 3 mm, screw diameter of 30 mm, and screw length of 742 mm. Following extrusion, the *Codonopsis pilosula* extrudates were dried in a forced-air drying oven at 70 °C for 6 h. A portion of the dried extrudates was subsequently milled and sieved again through an 80-mesh screen. The remaining extrudates were packaged in bags and stored in a freezer at −18 °C for subsequent use.

### 2.3. Preparation of Codonopsis pilosula Extract

Exactly 1 g of milled *Codonopsis pilosula* extrudate was accurately weighed and placed into a centrifuge tube. Then, 30 mL of 70% ethanol solution was added. The mixture was incubated in a 70 °C water bath for 45 min, followed by ultrasonic extraction in an ultrasonic bath at 380 W and 70 °C for 60 min. Subsequently, the mixture was centrifuged at 4000 rpm for 30 min. The resulting supernatant was collected as the extract solution for further use.

### 2.4. Determination of Polysaccharides in Codonopsis pilosula

Based on the method described by Liu et al. [14] with modifications, polysaccharides in *Codonopsis pilosula* extrudates were quantified using the phenol-sulfuric acid assay. Glucose standard solutions (0.2, 0.4, 0.6, 0.8, and 1.0 mL) were pipetted into test tubes and brought up to 1 mL with distilled water. Subsequently, 0.5 mL of 7% phenol solution was added precisely, followed by immediate addition of 2.5 mL concentrated sulfuric acid after thorough mixing. The reaction mixture was vortexed and allowed to stand for 30 min at 25 °C. A blank control was prepared identically without glucose standard solution. Absorbance was measured at 490 nm. The calibration curve was determined as Y = 0.5388X + 0.0045 (R^2^ = 0.9994). Following the same protocol, the absorbance of 1 mL *Codonopsis pilosula* extrudate extract was measured at 490 nm. Polysaccharide content was then calculated by interpolation from the calibration curve.

(1) $$Polysaccharide\;Content\;(mg/g)\;=\;M\;\times \;V\;\times \;(\frac{F}{m})$$

In the formula M = mass concentration of polysaccharides in the test sample calculated from the standard curve (mg/mL); V = volume of the test solution (mL); F = dilution factor; m = mass of the *Codonopsis pilosula* extrusion sample (g).

During the processing of *Codonopsis pilosula* using a twin-screw extruder, the barrel temperature, screw speed, and material moisture content were the three primary factors influencing its internal bioactive components. Single-factor experiments were conducted to investigate these factors. The extrusion process was initially performed with a barrel temperature of 130 °C, a screw speed of 200 rpm, and a material moisture content of 20%. Subsequently, the individual effects of barrel temperature (100 °C, 115 °C, 130 °C, 145 °C, 160 °C), screw speed (100 rpm, 150 rpm, 200 rpm, 250 rpm, 300 rpm), and material moisture content (18%, 20%, 22%, 24%, 26%) on the polysaccharide content of *Codonopsis pilosula* were examined.

Based on the single-factor experiments, screw speed (A), material moisture content (B) and barrel temperature (C) were selected as independent variables, coded and used to design a response surface experiment (RSM) using Design-Expert 8.0.6 software. This resulted in 17 experimental runs. The coded factor levels are presented in Table 1, and the corresponding RSM experimental design matrix is shown in Table 2.

### 2.5. Color

The L*, a* and b* values of the crushed and sieved samples (prepared per Section 2.2) were measured using a colourimeter to analyze chromaticity.

### 2.6. Water Solubility Index

An extruded Codonopsis sample (1 g, designated m_1_) was weighed into a centrifuge tube, mixed with 20 mL of distilled water, and shaken (110 rpm, 30 °C, 30 min) in a reciprocating thermostatic shaker bath. Following centrifugation (3000 rpm, 20 min), the supernatant was transferred to a pre-weighed evaporation dish (tare mass m_2_). The dish was dried to constant weight at 105 °C, and the final mass (designated m_3_) was recorded. The entire extraction and drying procedures were repeated three times, with average values calculated.

(2) $$Water\;Solubility\;Index\left(\%\right)\;=\;\frac{m_{3}\;-\;m_{2}}{m_{1}}\times 100\%$$

In the formula m_1_ denotes the mass of party ginseng extrudate (g), m_2_ denotes the mass of the net weight of the evaporating dish (g), m_3_ denotes the total mass of the dried dry matter and the evaporating dish (g).

### 2.7. Lobetyolin

The lobetyolin content in the extruded Codonopsis was determined using high-performance liquid chromatography [30]. Analysis was performed using an Agilent XDB-C18 column (4.6 mm × 200 mm, 5 μm)(Agilent, Santa Clara, CA, USA). The mobile phase consisted of acetonitrile (A) and 0.1% phosphoric acid in water (B), applied according to the gradient program detailed in Table 3. The column temperature was maintained at 30 °C, the flow rate was 0.8 mL/min and the detection wavelength was set at 232 nm. A standard stock solution of lobetyolin (0.5 mg/mL) was prepared in 75% methanol (designated as standard solution 6). Serial dilution was then performed: 3.0 mL of standard solution 6 was transferred to a 10 mL volumetric flask and diluted to volume with methanol to yield standard solution 5. This process was repeated stepwise to obtain standard solutions 4, 3, 2 and 1. Each standard solution was accurately loaded and 30 μL was injected into the HPLC system. This injection was performed in triplicate for each standard solution. A calibration curve was constructed by plotting the peak area (*Y*-axis) against the injected amount (*X*-axis). The resulting linear regression equation was Y = 1876.5X − 5.1753 (R^2^ = 0.9998). For sample preparation, extruded Codonopsis powder (1 g, sieved through an 80-mesh screen) was accurately weighed into a stoppered conical flask. Exactly 25 mL of 75% methanol was added, and the flask was weighed. The mixture was then sonicated for 45 min (450 W, 50 kHz). After sonication, the flask was cooled to room temperature, reweighed and any weight loss was replenished with 75% methanol. The mixture was shaken vigorously, filtered through a 0.45 μm membrane filter and the subsequent filtrate was collected as the test solution. The lobetyolin content in the sample solutions was calculated by substituting the measured peak areas into the regression equation.

### 2.8. Flavonoids

The total flavonoid content in the extruded Codonopsis was determined by ultraviolet spectrophotometry according to the method [31]. Standard curve preparation: aliquots (0.2, 0.4, 0.8, 1.2, 1.6 and 2.0 mL) of a 0.2 mg/mL rutin standard solution were pipetted into separate 15 mL centrifuge tubes. The volume in each tube was brought to 5 mL with 70% ethanol. Then, 0.3 mL of 5% NaNO_2_ solution was added to each tube. The mixtures were vortexed and incubated for 6 min. Subsequently, 0.3 mL of 10% Al(NO_3_)_3_ solution was added to each tube, followed by vortexing and a further 6 min incubation. Finally, 2 mL of 4% NaOH solution was added to each tube. After vortexing, the mixtures were incubated for 10 min. The absorbance of each solution was measured at 510 nm. A calibration curve was constructed using rutin as the standard. The resulting linear regression equation was Y = 0.5433X − 0.0065 (R^2^ = 0.9991), where Y represents absorbance and X represents rutin equivalent concentration (mg/mL). Sample Analysis: A 2.0 mL aliquot of the test Codonopsis extract solution was transferred to a 15 mL centrifuge tube and processed identically to the standard solutions described above.

### 2.9. Phenols

The total phenolic content in the *Codonopsis pilosula* extrudate was determined using the Folin–Ciocalteu colorimetric method [32]. Absorbed amounts (0.25 mL, 0.50 mL, 0.75 mL, 1.00 mL, 1.25 mL and 1.50 mL) of a 0.1 mg/mL gallic acid standard solution were separately pipetted into 15 mL centrifuge tubes. To each tube 1 mL of Folin–Ciocalteu reagent was added, followed by 2 mL of a 12% Na_2_CO_3_ solution. The mixture was thoroughly mixed and diluted to a final volume of 10 mL with deionized water. The reaction proceeded for 1 h at room temperature in the dark. The absorbance of each standard solution was measured at 765 nm. A standard calibration curve was constructed using gallic acid as the reference standard, yielding the regression equation: Y = 0.0069X + 0.0537 (R^2^ = 0.9981). For sample analysis, 1 mL of the sample extract solution was pipetted into a 15 mL centrifuge tube and processed identically to the above procedure. The absorbance of the sample extract was measured, and the total phenolic content was calculated based on the established standard curve.

### 2.10. DPPH

The DPPH radical scavenging assay was performed according to the method described in [33]. An 8.0 mg aliquot of DPPH was accurately weighed and dissolved in absolute ethanol, then diluted to a final volume of 100 mL with absolute ethanol. This DPPH stock solution was stored at 4 °C in the dark. For the assay, 1 mL of the *Codonopsis pilosula* extrudate extract solution was mixed with 0.25 mL of the DPPH solution in a test tube. The mixture was vortex-mixed thoroughly and incubated in the dark at room temperature for 60 min. The absorbance of this reaction mixture, A_i_, was measured at 517 nm using a UV-Vis spectrophotometer. The absorbance A_j_ was measured for a mixture containing 1 mL of *Codonopsis pilosula* extrudate extract solution and 0.25 mL of absolute ethanol. The radical blank control absorbance A_0_ was measured for a mixture of 0.25 mL DPPH solution and 1 mL absolute ethanol. The calculation formula is as follows:

(3) $$DPPH\;radical\;scavenging\;activity\left(\%\right)\;=\;\left(1-\frac{A_{i}-A_{j}}{A_{0}}\right)\times 100\%$$

In the formula A_i_ denotes the absorbance value of Codonopsis extrudate extract; A_j_ denotes the absorbance value of blank Codonopsis extrudate extract; A_0_ denotes the absorbance value of DPPH control.

### 2.11. Hydroxyl Radical

The hydroxyl radical-scavenging activity was determined according to the method of Atere et al. [34] with slight modifications. Briefly, 2 mL of the extract was transferred into a test tube, followed by the successive addition of 2 mL of 9 mmol/L ferrous sulfate solution and 2 mL of 9 mmol/L salicylic acid in ethanol. Then, 2 mL of 8.8 mmol/L hydrogen peroxide solution was added to initiate the reaction. The mixture was incubated in a water bath at 37 °C for 30 min. The absorbance (A_x_) was measured at 510 nm using a UV spectrophotometer. A blank control (A_0_) was prepared by replacing the Codonopsis extract with an equal volume of distilled water.

(4) $$Hydroxyl\;radical\;scavenging\;activity\left(\%\right)\;=\;\frac{A_{0}-A_{x}}{A_{0}}\times 100\%$$

In the formula A_x_ denotes the absorbance of the extract of Codonopsis extrudate; A_0_ denotes the absorbance of the blank control in distilled water.

### 2.12. ABTS^+^

The ABTS^+^ radical scavenging capacity of *Codonopsis pilosula* extrusion extract was assessed using a modified method of Fernando et al. [35]. The ABTS^+^ stock solution was prepared by mixing equal volumes of 7 mmol/L ABTS^+^ and 2.45 mmol/L potassium persulfate solutions. This mixture was wrapped in aluminum foil, stored at room temperature in the dark for 16 h and diluted with absolute ethanol to an absorbance of 0.70 ± 0.02 at 734 nm prior to use. Aliquots (0.3 mL) of sample solutions at varying concentrations (0.1–2 mg/mL) were mixed with 3 mL of ABTS^+^ working solution. After incubation at room temperature for 6 min, the absorbance A_1_ was immediately measured at 734 nm. The ABTS^+^ scavenging rate was calculated as follows:

(5) $${ABTS}^{+}\;radical\;scavenging\;activity\left(\%\right)\;=\;\frac{A_{0}-\left(A_{1}-A_{2}\right)}{A_{0}}\;\times \;100\%$$

In the formula A_1_ denotes the absorbance of the sample set; A_2_ denotes the absorbance of 95% ethanol solution instead of ABTS^+^ working solution; A_3_ denotes the absorbance of 0.3 mL of 95% ethanol solution instead of the sample solution.

### 2.13. α-Glucosidase Inhibitory Rate

A 5 mmol/L solution of PNPG and a 0.1 U/mL solution of α-glucosidase were prepared separately in 100 mmol/L sodium phosphate buffer (pH 6.8). 50 μL of sample solutions at various concentrations (0.1–2 mg/mL) were pipetted into wells of a 96-well plate. Then, 50 μL of the PNPG solution was added to each well. The mixture was pre-incubated at 37 °C for 10 min. Subsequently, 50 μL of the α-glucosidase solution was added, and the reaction mixture was incubated at 37 °C for 30 min. The reaction was terminated by adding 50 μL of 0.1 mol/L Na_2_CO_3_ solution. The absorbance was measured at 409 nm [36]. The calculation formula is as follows:

(6) $$\alpha -Glucosidase\;inhibition\;rate\left(\%\right)\;=\;\;\left(\frac{A_{2}-A_{1}}{A_{2}-A_{0}}\right)\;\times \;100\%$$

In the formula A_0_ = Absorbance of the blank control; A_1_ = Absorbance of the test sample; A_2_ = Absorbance of the negative control.

### 2.14. Development of Codonopsis pilosula-Oat Powder

Process Flow: Codonopsis powder → Mixed with water → Twin-screw extrusion → Hot air drying → Natural cooling → Pulverization through an 80-mesh sieve → Store in a plastic bag in the refrigerator → Proportional mixing of excipients → Codonopsis-oat powder.

The extruded *Codonopsis pilosula* powder constituted the primary ingredient of the Codonopsis-oat powder, with a fixed dosage of 10 g per serving based on the permitted usage range specified in the Chinese Pharmacopoeia. By adjusting the addition levels of supplementary ingredients (oat powder, red date powder, maltodextrin), the mixture was thoroughly blended and prepared with hot water (above 80 °C). Using sensory evaluation as the indicator, we investigated the effects of oat powder, red date powder and maltodextrin addition levels, respectively, on the sensory quality of *Codonopsis pilosula* oat powder. Using single-factor experiments, we established the addition level ranges for the three supplementary ingredients, as detailed in Table 4. Subsequently, based on the single-factor experiments, we selected the addition levels of oat powder (A), red date powder (B), maltodextrin (C), and error estimation (D). Using sensory evaluation as the indicator, an L9(3^4^) orthogonal array design with a dummy column was employed. The orthogonal experimental design is presented in Table 5, and the sensory scoring protocol referenced the method of Alemayehu et al. [37] shown in Table 6.

### 2.15. Statistical Analysis

All experiments were independently repeated at least three times. Origin 2019, SPSS 17.0 (Duncan’s multiple range test) and Design Expert 8.0.6 software were used to analyze and process the data. Data are presented as means ± standard deviations. Statistical significance was considered at *p* < 0.05.

## 3. Results

### 3.1. Single-Factor Results of Codonopsis Polysaccharides

**Figure 1:**
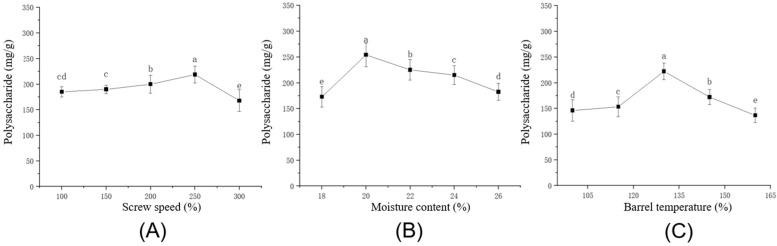
Effects of (**A**) Screw Speed, (**B**) Moisture Content, (**C**) Barrel Temperature on Polysaccharide Content. Different lowercase letters within each subfigure indicate significant differences (*p* < 0.05) among the groups.

Figure 1A demonstrates that polysaccharide content exhibited an initial decrease, followed by an increase and subsequent decline as screw speed increased. The maximum polysaccharide content occurred at 250 rpm. Polysaccharide yield was significantly influenced by screw speed, though minimal variation occurred between 100–200 rpm. Elevated screw speeds intensified shear force and friction, inducing complete cell wall disruption and enhanced release of soluble compounds. This explains the peak polysaccharide content at 250 rpm. However, excessive speeds (300 rpm) reduced material residence time, impairing cell wall fragmentation and limiting polysaccharide extraction [38]. Consequently, single-factor experiments determined 250 rpm as the optimal screw speed, with an effective operational range of 200–300 rpm.

Figure 1B demonstrates that polysaccharide content peaked at 20% material moisture content and reached its minimum at 18%. A progressive increase in moisture content initially elevated polysaccharide levels, followed by a gradual decline. Processing at moisture content (≤18%) generated excessive barrel friction and shear force, inducing macromolecular degradation and reduced polysaccharide yield. Preliminary trials at 17% moisture content caused complete extruder blockage, halting processing. Consequently, a moisture content of 18% was established as the minimum viable threshold. The optimal extraction yield was achieved at 20% moisture content. At this optimal level, moisture acts as a plasticizer and lubricant, effectively reducing barrel friction and moderating the specific mechanical energy input and shear force [39]. This balanced mechanical action facilitates effective cell wall disruption while minimizing the degradation and loss of material due to excessive shear. Conversely, when the moisture content exceeds this optimum, the material transitions into an overly fluid-like state. This excessive lubrication severely diminishes the transfer of shear energy from the screws to the material, thereby impairing the mechanical disruption of cell walls and the subsequent release of bioactive compounds [40,41]. Based on these single-factor experiments, a moisture content of 20% was identified as ideal, with a practicable operational range of 18–22%.

Figure 1C indicates that polysaccharide content initially increased and then decreased with rising barrel temperature, peaking at 130 °C. In the range of 100–130 °C, polysaccharide yield increased progressively, likely because the enhanced extractability due to heat-induced cell wall breakdown outweighed any potential degradation. At 130 °C, maximal polysaccharide extraction was achieved. Beyond this point, the significant decline in polysaccharide content is primarily due to the thermal decomposition of the polysaccharides themselves and the intensification of Maillard reactions, which consume reducing sugars and are amplified by the combined thermal and mechanical energy input [42]. This contrasts with reported starch degradation under high pressure and shear [43], which may increase reducing sugar levels. Single-factor optimization thus established 130 °C as optimal, with an operational range of 115–145 °C.

### 3.2. Codonopsis pilosula Polysaccharide

As shown in Supplementary Table S1, the effects of various factors on the polysaccharide content of *Codonopsis pilosula* extrudate were ranked as C > B > A. The barrel temperature had a highly significant effect on the polysaccharide content of *Codonopsis pilosula* extrudate (*p* < 0.01), the moisture content of the material had a significant effect (*p* < 0.05) and the screw speed had no significant effect (*p* > 0.05). The interaction term AC had an extremely significant effect on the polysaccharide content of *Codonopsis pilosula* extrudate (*p* < 0.01), indicating that the interaction effect between barrel temperature and screw speed was significant.

**Figure 2:**
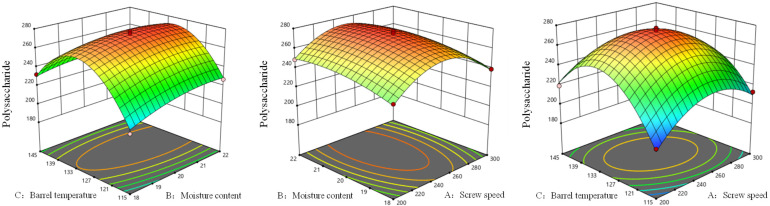
Response surface analysis of extrusion processing parameters on polysaccharide yield of *Codonopsis pilosula*.

**Figure 3:**
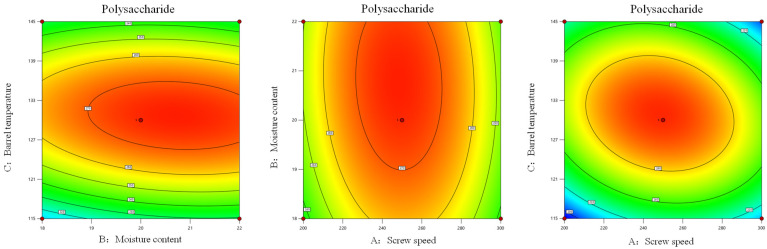
Contour analysis of extrusion processing parameters on polysaccharide yield of *Codonopsis pilosula*.

As shown in Figure 2 and Figure 3, the polysaccharides of AB, AC and BC all exhibited a trend of first increasing and then decreasing. The response surface plot showed that the change in AC was the most obvious, so the interaction of AC had a stronger effect on the polysaccharides of AB and BC. The contour plot shows that the AC curve was evenly distributed, elliptical in shape and with obvious color changes. Through a comparison of synergistic effects, it could be seen that AC > BC > AB.

The content of *Codonopsis polysaccharides* significantly increased from 244.41 mg/g in the raw material to a maximum of 277.25 mg/g following extrusion, representing a net gain of 32.84 mg/g. This enhancement can be primarily attributed to the intense mechanical and thermal forces experienced during twin-screw extrusion. The combined action of high shear, temperature, and pressure effectively disrupts the rigid plant cell walls, thereby facilitating the release of bound and intracellular polysaccharides into an extractable form [42]. Concurrently, the high-shear environment induces the depolymerization of larger, insoluble polysaccharide molecules into smaller, soluble fragments. This mechanical breakdown, followed by possible in situ reorganization, contributes to the measured increase in soluble polysaccharide content [44]. However, as the extrusion temperature surpasses the optimal point, the polysaccharide content begins to decline. This decrease is likely a consequence of thermal degradation and the intensification of the Maillard reaction between reducing sugars and amino acids, both of which are promoted by excessive thermal energy [45].

### 3.3. Water Solubility Index

As shown in Supplementary Table S1, the influence of various factors on the water solubility index of *Codonopsis pilosula* extrudate was ranked as A > B > C. Both screw speed and material moisture content had a significant effect on the water solubility index of *Codonopsis pilosula* extrudate (*p* < 0.05), while barrel temperature had no significant effect on the water solubility index of *Codonopsis pilosula* extrudate (*p* > 0.05). The interaction terms AB, AC and BC all had no significant effect on the water solubility index of *Codonopsis pilosula* extrudate (*p* > 0.05), indicating that there was no significant interaction among the three factors.

**Figure 4:**
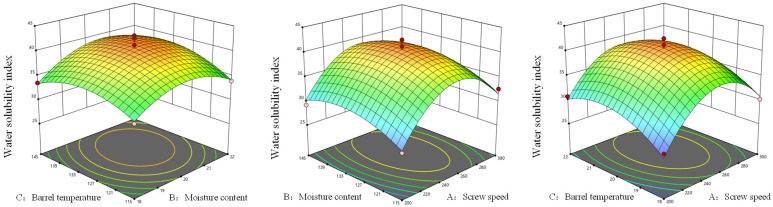
Response surface analysis of extrusion processing parameters on water solubility index of *Codonopsis pilosula*.

**Figure 5:**
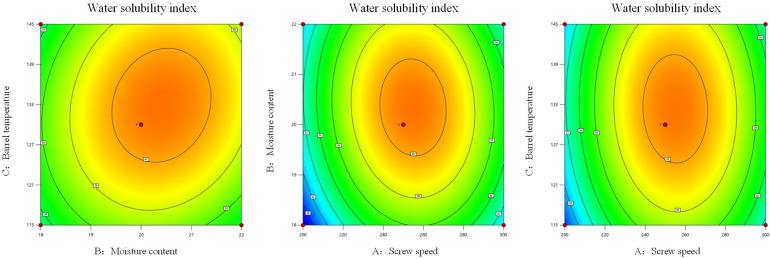
Contour analysis of extrusion processing parameters on water solubility index of *Codonopsis pilosula*.

As shown in Figure 4 and Figure 5, the results of the synergistic effects between various factors could be observed based on the response surface plot and contour plots. In the response surface plot, the interaction effects of factors AB, AC, and BC on the water solubility index of *Codonopsis pilosula* exhibited a trend of first increasing and then decreasing. The response surface of AB was steeper than that of AC, indicating that the interaction effects of these two factors had a greater influence on the water-soluble indicators of *Codonopsis pilosula*. In the contour plot, the factors AB and AC were uniformly distributed along the axial contour lines, with the contour lines forming nearly circular shapes, indicating that the synergistic effects of screw speed and material moisture content, as well as screw speed and barrel temperature, had a negligible impact on the water-soluble index. The factor BC was uniformly distributed along the axial contour lines, with the contour lines forming elliptical shapes, indicated that the synergistic effect of BC had a stronger impact on the water-soluble index than that of AB and AC. Based on the comparison of synergistic effects, BC > AB > AC, which was consistent with the results of the analysis of variance. After double-screw extrusion, the water solubility index of *Codonopsis pilosula* increased from the original 12.99% to a maximum of 42.53%, representing an increase of 29.54%. The increase in the water-soluble index might be attributed to the fact that during the extrusion process, the material was subjected to high temperature, high pressure and high shear force, which caused the breakdown of *Codonopsis pilosula* cell walls and an increase in soluble proteins and carbohydrates. Simultaneously, large-molecule substances such as proteins and carbohydrates were released, and high shear force caused them to degrade into smaller molecules, resulting in an increase in hydrophilic substances and thereby elevating the water-soluble index [46].

### 3.4. Color

The L* value indicates the lightness of a color. In food processing, a decrease in L* value is typically associated with browning reactions, such as the Maillard reaction, caramelization, and enzymatic browning. After twin-screw extrusion processing, the L* value of *Codonopsis pilosula* decreased from an initial value of 66.29 to a minimum of 58.75, corresponding to a reduction of 7.54. This decrease was likely due to the Maillard reaction, which produced dark-colored compounds. Additionally, the instantaneous evaporation of moisture during extrusion resulted in a darker surface appearance, further contributing to the reduction in L* value. The a* value represents the red-green axis, while the b* value corresponds to the yellow-blue axis. Maillard and caramelization reactions produce dark brown substances with reddish-brown and yellowish-brown tones, leading to increases in both a* and b* values. After extrusion, the a* value of *Codonopsis pilosula* increased from 6.03 to a maximum of 11.38—an increase of 5.53—while the b* value increased from 23.58 to 31.28, an increase of 7.7. These increases were attributed to the formation of brown pigments through the Maillard reaction, as well as the disruption of native pigment stability under high temperature and pressure, resulting in significant changes in a* and b* values.

### 3.5. Lobetyolin

Lobetyolin is a characteristic bioactive polyacetylene compound in *Codonopsis pilosula*. As a thermolabile constituent, lobetyolin exhibits sensitivity to temperature, light, oxygen, and mechanical stress during processing [47]. Following extrusion processing, the lobetyolin content decreased from an initial 360.53 μg/g to a maximum post-extrusion level of 273.12 μg/g, representing a reduction of 87.41 μg/g. This degradation might be attributed to the inherent instability of polyacetylene and enyne bonds within its molecular structure, which were prone to cleavage, rearrangement or decomposition under thermal stress. Concurrently, moisture evaporation during extrusion under high temperature and pressure conditions likely accelerated hydrolytic degradation of lobetyolin. Notably, despite the observed reduction, the high-temperature short-time nature of extrusion processing limited thermal exposure duration, thereby enhancing lobetyolin retention compared to conventional thermal treatments [39].

### 3.6. Flavonoids

Flavonoids in *Codonopsis pilosula* are one of its important bioactive components, exhibiting antioxidant, anti-inflammatory, immune-modulating, cardiovascular protective and neuroprotective effects. The total flavonoid content of *Codonopsis pilosula* decreased from 18.89 mg/g to a maximum of 18.74 mg/g after extrusion, representing a reduction of 0.15 mg/g. The change in total flavonoid content was not statistically significant (*p* < 0.05). The reason for the non-significant change in flavonoids in *Codonopsis pilosula* might be that the high temperature, high pressure and high shear force during the pressing process effectively disrupt the structure of plant cell walls. This may have resulted in the release of flavonoids from their originally bound form into a free or more readily extractable state [48]. This release effect may partially offset the losses caused by minor thermal degradation, resulting in a non-significant decrease in total flavonoid content. Although most flavonoids are heat-sensitive, their stability varies among different types. The main flavonoid monomers in *Codonopsis pilosula*, such as luteolin and luteolin glycoside, exhibit relatively good thermal stability under short-term high temperature conditions [49].

### 3.7. Phenols

Total phenolic content is typically a key indicator for evaluating the antioxidant activity of *Codonopsis pilosula*. Higher content generally indicates stronger potential antioxidant, anti-ageing and disease-resistant properties, such as cardiovascular diseases and certain cancers [50]. After twin-Screw extrusion, the total phenolic content, which was originally 4.62 mg/g, reached a maximum of 5.77 mg/g, representing an increase of 1.15 mg/g in the total phenolic content of *Codonopsis pilosula*. The increase in total phenolic content might be attributed to the fact that a significant portion of the phenolic compounds in *Codonopsis pilosula* existed in a bound state. The intense shear force, high temperature and high pressure during twin-screw extrusion effectively disrupted the cell wall structure of *Codonopsis pilosula*, broke down these physical barriers and chemical bonds to release the bound phenolic compounds, transforming them into free or more easily extractable forms [51]. Additionally, high temperature, pressure and shear force might promote partial degradation of some complex macromolecular substances in *Codonopsis pilosula* [52]. This degradation might produce smaller-molecular-weight compounds with phenolic hydroxyl groups, ultimately leading to an increase in total phenolic content after extrusion processing.

### 3.8. Antioxidant Properties

As a traditional Chinese medicine and food ingredient, one of the important bioactive properties is antioxidant capacity of *Codonopsis pilosula*, which helps protect cells and tissues from oxidative damage [53]. To evaluate the antioxidant properties of *Codonopsis pilosula*, the DPPH, ABTS^+^ and hydroxyl radical scavenging rates were measured before and after squeezing. After extrusion processing, the DPPH radical scavenging rate of *Codonopsis pilosula* increased from 60.43% to a maximum of 73.19% after extrusion, showing a significant increase in DPPH radical scavenging rate. While the ABTS^+^ radical scavenging rate showed a slight increase from 83.88% to a maximum of 87.89% after extrusion. The hydroxyl radical scavenging rate increased from 52.89% to a maximum of 74.09% after extrusion, indicating a significant improvement in hydroxyl radical scavenging activity. Overall, extrusion processing could significantly enhance the in vitro antioxidant activity of *Codonopsis pilosula*. The potential reason for the increased antioxidant properties of *Codonopsis pilosula* might be due to the increased content of natural compounds related to antioxidant activity, such as phenolic compounds and polysaccharides, within the extrusion processing, as well as the degradation of large-molecule antioxidant substances into smaller fragments or oligomers under the influence of extrusion shear force, which might confer higher antioxidant activity [54].

### 3.9. α-Glucosidase Inhibitory Rate

Inhibiting α-glucosidase is currently recognised as one of the most widely studied mechanisms for reducing postprandial hyperglycaemia. The α-glucosidase inhibition rate is a core functional indicator for assessing the potential of *Codonopsis pilosula* to regulate carbohydrate digestion and lower postprandial blood glucose levels [55]. The higher the inhibition rate, the stronger the potential anti-diabetic effect. After extrusion, the α-glucosidase inhibition rate of *Codonopsis pilosula* increased from 65.38% to a maximum of 82.32%, significantly enhancing its hypoglycaemic activity. This might be attributed to the powerful shear force and pressure during extrusion, which effectively disrupts the cell wall structure of *Codonopsis pilosula*. Active components originally enclosed within cells or tightly bound to the cell wall matrix, such as saponins, flavonoids and certain polysaccharides, are more fully released [56]. These active components can more effectively interact with α-glucosidase and perform their functions, thereby increasing the α-glucosidase inhibition rate.

### 3.10. Optimal Process

For the subsequent development of *Codonopsis pilosula*-related food products, data were analyzed and processed using Design-Expert 8.0.6 software to optimize parameters for achieving the highest values of water solubility index and *Codonopsis pilosula* polysaccharide content. The optimal process conditions for twin-screw extrusion of codonopsis were determined to be screw speed of 252.07 rpm, material moisture content of 20.45% and barrel temperature of 131.31 °C. The predicted theoretical values for codonopsis polysaccharides and water solubility index based on the regression model were 274.64 mg/g and 40.84%, respectively. Based on actual experimental operations, the experimental conditions selected were screw speed of 250 rpm, moisture content of 20%, and barrel temperature of 131 °C, with three replicate experiments conducted. The average polysaccharide content from the three experiments was 271.00 mg/g, differing by 3.64 mg/g from the theoretically predicted value. The average water solubility index of the three experiments was 40.79%, differing by 0.05% from the theoretically predicted value. This indicated that the model could effectively simulate and predict the processing of *Codonopsis pilosula* using a twin-screw extrusion process and that twin-screw extrusion significantly increased the polysaccharide content and water solubility index of *Codonopsis pilosula* (*p* < 0.05). The relevant indicators of *Codonopsis pilosula* under optimal process conditions are shown in Table 7.

### 3.11. Codonopsis Oat Powder

The formulation development of the instant Codonopsis powder involved the incorporation of oat powder, red date powder, and maltodextrin. Oat powder was chosen for its richness in soluble dietary fiber, which aids in regulating blood sugar levels and reducing postprandial blood glucose and insulin responses [57]. Red date powder served as a natural sweetener to improve flavor, while maltodextrin acted as a processing aid to enhance the powder’s solubility and physical stability. The objective of adding these ingredients was to improve the palatability of Codonopsis and to advance its processing for broader food applications. This section aims to optimize the addition levels of these three key components through sensory evaluation.

**Figure 6:**
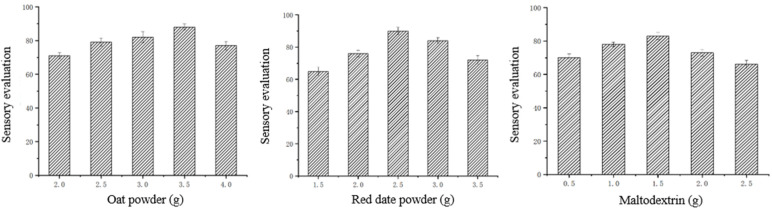
Effects of oat flour, red date powder and maltodextrin concentrations on sensory profile of *Codonopsis pilosula* oat powder.

As shown in Figure 6, as the amount of oat flour added increased, the sensory score first rose and then decreased. When the oat flour was added in amount less than 3.5 g, the Codonopsis oat flour mixture became increasingly viscous from thin to thick, the solution color gradually lightened from red, and the Codonopsis flavour was relatively strong. The viscosity was moderate, and the texture was great when the added amount was 3.5 g. It had a unique flavour and the sensory score was the highest. When less than 3.5 g of oat flour was added, the Codonopsis-oat flour mixture exhibited a gradual increase in viscosity, a lightening of color, and a relatively strong Codonopsis flavor. As the amount of red date powder added increased, the sensory score tended to rise and then fell. When the amount of oat flour added was less than 2.5 g, the color of the Codonopsis oat flour gradually faded after mixing, the Codonopsis flavour gradually faded, the sweetness gradually increased, and the sensory score gradually rose. The viscosity was moderate, the color was appropriate, and the texture was optimal when the added amount was 2.5 g. It had a unique flavour and the sensory score was highest. When the red date powder addition exceeded 2.5 g, the texture deteriorated, the color gradually darkened, the Codonopsis flavour diminished, and the sweetness became overly sweet, leading to a poorer sensory score. Maltodextrin is a widely used food ingredient characterized by its slight sweetness, ability to reduce browning, and high stability owing to its resistance to caking and moisture absorption. As the amount of maltodextrin added increased, the sensory score tended to rise and then fall. When the amount of maltodextrin added was less than 1.5 g, Codonopsis oat flour had fewer lumps, a lighter taste, lower viscosity and the sensory score gradually increased. The viscosity was moderate, and the taste was great when the added amount was 1.5 g. There were fewer lumps, and the sensory score was the highest. When the maltodextrin addition exceeded 1.5 g, the taste gradually deteriorated, lumps increased, and the mixture became more viscous when combined with oatmeal powder, leading to a decline in sensory scores.

The results of the orthogonal experiment are shown in Table 8. Based on the R values, it could be seen that B > A > C > D (red date powder > oat flour > maltodextrin > blank column). Factors A, B and C were all greater than the blank column, so all three factors were major factors. Based on the sizes of factors K_1_, K_2_ and K_3_, it could be seen that A_2_ > A_3_ > A_1_, B_2_ > B_3_ > B_1_ and C_2_ > C_3_ > C_1_. The optimal ratio was A_2_B_2_C_2_. Among the nine test groups, test group 5 had the same ratio as the optimal ratio, with the highest sensory score of 88.7 points. The optimal factor ratio for Codonopsis oat flour was 10 g of squeezed Codonopsis powder, 3.5 g of oat flour, 2.5 g of red date powder, and 1.5 g of maltodextrin.

## 4. Conclusions

This study successfully developed an instant edible powder using *Codonopsis pilosula* as the primary raw material, with extrusion technology employed as a pretreatment to enhance its functional properties. The optimal extrusion parameters, determined by Response Surface Methodology, were as follows: screw speed of 250 rpm, moisture content of 20%, and barrel temperature of 131 °C. Under these conditions, the water solubility index and polysaccharide content of the Codonopsis were significantly increased. Concurrently, notable enhancements were observed in its antioxidant activity and α-glucosidase inhibitory activity. These results confirm that extruded Codonopsis is a suitable ingredient for instant food products. The marked increase in the release of bioactive compounds, coupled with its postprandial blood glucose-lowering potential, highlights its significant application prospects in the functional food industry.

The increase in water solubility index following extrusion greatly improved the dispersibility of the Codonopsis powder in water, which is critical for its application as an instant beverage. The concurrent enhancement of its internal effective components further elevates the application value of Codonopsis as a food ingredient. Extrusion processing, characterized by its short processing time, high production efficiency, and continuous operation, offers considerable advantages for industrial food production. For Codonopsis, efficiently increasing its active components and improving solubility are essential prerequisites for the large-scale manufacturing of instant powder products. This study provides a systematic approach by integrating the optimized extrusion parameters identified in the initial stage with the subsequent development of the optimal powder formulation. This integrated strategy paves the way for the streamlined industrial production of Codonopsis-based foods and offers a theoretical foundation for related food manufacturing. Future research should focus on low-temperature extrusion strategies to better retain heat-sensitive compounds and maintain the product’s original color and flavor profile.

## Figures and Tables

**Table 1:** Experimental Factor Level Design.

Coded Levels	Factors		
	A—Screw Speed (rpm)	B—Moisture Content (%)	C—Barrel Temperature (°C)
−1	200	18	115
0	250	20	130
1	300	22	145

**Table 2:** Response surface experimental design scheme.

Test Number	Factors		
	A—Screw Speed (rpm)	B—Moisture Content (%)	C—Barrel Temperature (°C)
1	200	22	130
2	300	20	115
3	250	22	145
4	250	18	115
5	250	20	130
6	200	20	115
7	300	22	130
8	300	18	130
9	250	22	115
10	200	20	145
11	250	20	130
12	300	20	145
13	200	18	130
14	250	20	130
15	250	18	145
16	250	20	130
17	250	20	130

**Table 3:** Mobile phase elution procedure.

Time (min)	Mobile Phase A (%)	Mobile Phase B (%)
0 → 20	10	90
20 → 30	10 → 30	90 → 70
30 → 50	30 → 70	70 → 30

**Table 4:** Single-factor test table for *Codonopsis pilosula*-oat powder.

Factors	Coded Levels				
	1	2	3	4	5
Oat flour (g)	2.0	2.5	3.0	3.5	4.0
Red date powder (g)	1.5	2.0	2.5	3.0	3.5
Maltodextrin (g)	0.5	1.0	1.5	2.0	2.5

**Table 5:** Orthogonal experimental design factors and level values for *Codonopsis pilosula*-oat powder.

Coded Levels	Factors			
	A Oat Flour/g	B Red Date Powder/g	C Maltodextrin/g	D Error Estimation
1	3.0	2.0	1.0	/
2	3.5	2.5	1.5	/
3	4.0	3.0	2.0	/

**Table 6:** Sensory rating scale.

Attributes	Characteristics	Standard/Points
Color	Bright and vivid colors	8–10
	Dull color	5–7
	Color abnormalities, browning	1–4
Smell	With the aroma of codonopsis and red date	18–20
	No noticeable scent	8–17
	Off-flavors	1–7
Taste	Moderately sweet, with a distinct flavour of the original ingredients	27–30
	The sweetness is too strong or too weak and the taste of the raw materials is quite noticeable	11–26
	Too sweet or too bland, with the flavours of the ingredients not coming through clearly	1–10
Solubility	Easily dissolves after mixing, no lumps	18–20
	Easily dissolves after mixing, with minimal clumping	8–17
	Difficult to dissolve after mixing, with a large amount of clumping	1–7
Organisational status	Viscous paste-like consistency and good flowability	18–20
	Slightly layered, relatively thick or relatively thin	8–17
	Severe stratification, too thick or too thin	1–7

**Table 7:** Comparison of the composition of *Codonopsis pilosula* before and after extrusion.

Indicator	Before Extrusion	After Extrusion
Water solubility index (%)	12.99 ± 0.07	40.79 ± 0.11 *
Color L*	66.29 ± 0.03	53.46 ± 0.06 *
Color a*	6.03 ± 0.08	8.48 ± 0.07 *
Color b*	23.58 ± 0.13	28.54 ± 0.11 *
Polysaccharide (mg/g)	244.41 ± 2.33	271.00 ± 3.01 *
Total flavonoids (mg/g)	18.89 ± 0.03	15.46 ± 0.07 *
Total phenols (mg/g)	4.62 ± 0.04	4.42 ± 0.09
DPPH (%)	60.43 ± 0.17	67.35 ± 0.21 *
ABTS^+^ (%)	83.88 ± 0.25	84.11 ± 0.19
Hydroxyl radical (%)	52.89 ± 0.27	69.27 ± 0.13 *
α-Glucosidase inhibition rate (%)	65.38 ± 0.12	64.95 ± 0.15
Lobetyolin (μg/g)	360.53 ± 1.21	204.13 ± 2.68 *

Note: Data are presented as mean ± standard deviation (*n* = 3). Values in the same row followed by * indicate significant differences (*p* < 0.05) among the data, determined by Duncan’s multiple range test.

**Table 8:** Data of orthogonal test of *Codonopsis pilosula* oat powder.

Number	A Oat Flour/g	B Red Date Powder/g	C Maltodextrin/g	D (Empty Column)	Rating
1	3.0	2.0	1.0	1	75.8
2	3.0	2.5	2.0	2	85.3
3	3.0	3.0	1.5	3	79
4	3.5	2.0	2.0	3	83.5
5	3.5	2.5	1.5	1	88.7
6	3.5	3.0	1.0	2	83.6
7	4.0	2.0	1.5	2	78.3
8	4.0	2.5	1.0	3	83.5
9	4.0	3.0	2.0	1	83.7
K1	240.1	237.6	242.9	248.2	
K2	255.8	257.5	252.5	247.2	
K3	245.5	246.3	246.0	246.0	
k1	80.0	79.2	81.0	82.7	
k2	85.3	85.8	84.2	82.4	
k3	81.8	82.1	82.0	82.0	
R	5.2	6.6	3.2	0.7	
Comparison of factor sizes: B > A > C > D				Optimal ratio: A2B2C2	

## Data Availability

The raw data supporting the conclusions of this article will be made available by the authors on request.

## Supplementary Materials

The following supporting information can be downloaded at: https://www.mdpi.com/article/10.3390/foods14203485/s1, Table S1: ANOVA of regression models for chemical indicators in extruded *Codonopsis pilosula*.
