# Supplementary material for: Enhancement of Bioactive Compounds and Antioxidant Properties in Codonopsis pilosula Through Extrusion Processing and Development of Codonopsis-Oat Powder
Source: Foods. 2025 Oct 13;14(20):3485. doi: 10.3390/foods14203485 (PMC12563478; doi:10.3390/foods14203485)
Supplement: Supplementary file 1 [file foods-14-03485-s001.zip › foods-3884628-supplementary.pdf]

Table S1. ANOVA of regression models for chemical indicators in extruded Codonopsis pilosula.

Sources of variance	Polysaccharide		Flavonoid		Phenol		Lobetyolin		Water solubility index	
	P value	Significant	P value	Significant	P value	Significant	P value	Significant	P value	Significant
Model	< 0.0001	**	0.0019	**	0.0094	**	0.004	**	0.0002	**
A	0.5336		0.4207		0.0014	**	0.1259		0.0124	*
B	0.0181	*	0.2961		0.2202		0.0123	*	0.0295	*
C	0.0094	**	0.8150		0.0495	*	0.0003	**	0.1031	
AB	0.4619		0.0705		0.7538		0.6005		0.3410	
AC	0.0027	**	0.3583		0.1231		0.222		0.6537	
BC	0.1087		0.0409	*	0.4321		0.0424	*	0.2755	
Lack of fit	0.2279		0.1956		0.4411		0.2236		0.5228	

Note: ** $P < 0.01$ (highly significant); * $P < 0.05$ (significant).
